# Parental experiences of providing skin-to-skin care to their newborn infant—Part 2: A qualitative meta-synthesis

**DOI:** 10.3402/qhw.v9.24907

**Published:** 2014-10-13

**Authors:** Agneta Anderzén-Carlsson, Zeni C. Lamy, Maria Tingvall, Mats Eriksson

**Affiliations:** 1 Centre for Health Care Sciences, Örebro University Hospital, Örebro, Sweden; 2 School of Health and Medical Sciences, Örebro University, Örebro, Sweden; 3 Departamento de Saúde Pública, Universidade Federal do Maranhão, Hospital Universitário, Sao Luis, Brazil; 4 Department of Obstetrics and Gynaecology, Örebro University Hospital, Örebro, Sweden

**Keywords:** Kangaroo mother care, meta-study, meta-synthesis, newborn infant, skin-to-skin care, qualitative research

## Abstract

**Aim:**

To synthesize and interpret qualitative research findings focusing on parental experiences of skin-to-skin care (SSC) for newborn infants.

**Background:**

SSC induces many benefits for newborn infants and their parents. Three meta-analyses have been conducted on physiological outcomes, but no previous qualitative meta-synthesis on parental experiences of SSC has been identified.

**Design:**

The present meta-synthesis was guided by the methodology described by Paterson and co-workers.

**Data sources:**

Four databases were searched, without year or language limitations, up until December 2013. Manual searches were also performed. The searches and subsequent quality appraisal resulted in the inclusion of 29 original qualitative papers from 9 countries, reporting experiences from 401 mothers and 94 fathers.

**Review methods:**

The meta-synthesis entails a meta-data analysis, analysis of meta-method, and meta-theory in the included primary studies. Based on the three analyses, the meta-synthesis represents a new interpretation of a phenomenon. The results of the meta-data analysis have been presented as a qualitative systematic review in a separate paper.

**Results:**

When synthesizing and interpreting the findings from the included analyses, a theoretical model of *Becoming a parent under unfamiliar circumstances* emerged. Providing SSC seems to be a restorative as well as an energy-draining experience. A supportive environment has been described as facilitating the restorative experience, whereas obstacles in the environment seem to make the provision of SSC energy-draining for parents. When the process is experienced as positive, it facilitates the growth of parental self-esteem and makes the parents ready to assume full responsibility for their child.

**Conclusion:**

The results show that SSC can be interpreted not only as a family-including and important health care intervention but also in terms of actually becoming a parent. The process of becoming a parent in this specific situation is influenced by external factors in three different levels; family and friends, community, and society at large. The descriptions of providing SSC are similar to what has previously been described as the natural process of becoming a mother or a father.

Skin-to-skin care (SSC) between newborn infants and their parents has been found to have many beneficial effects for the newborns and their parents (Conde-Agudelo, Belizán, & Diaz-Rossello, 2011) and is recommended in all contexts, from low-resource settings, to high-tech neonatal intensive care units (NICU). A large body of research has focused on the physiological and medical effects of SSC. Other studies have explored parental experiences of SSC for their newborn infant, but to date there is no unified picture of their findings. This meta-synthesis synthesizes and interprets the findings from 29 original research papers from different countries and clinical settings. The detailed findings of the meta-analysis step are presented in a qualitative systematic review (Anderzén-Carlsson, Lamy, & Eriksson, 2014). The focus of the present paper is to describe the theoretical model that emerged from the meta-synthesis.

SSC or Kangaroo Mother Care (KMC) started as a way to deal with a shortage of incubators in an overcrowded Colombian neonatal unit, leading to less morbidity and less infant abandonment in the preterm infant population (Charpak et al., 2005). Original components of KMC were the kangaroo position, exclusive breastfeeding, and early discharge. Since the initiation of SSC/KMC, there have been shifts in the paradigm: 1) from an alternative method for the care of delicate infants in low-resource settings to neonatal care worldwide, 2) from being used continuously to shorter periods during the day (Nyqvist et al., 2010), and 3) from a method for use with preterm or low birth weight infants to being regarded as equally beneficial for healthy newborns (Moore, Anderson Gene, & Bergman, 2007). Today, SSC/KMC is recommended by the World Health Organisation (2003), professional organizations (Council of International Neonatal Nurses, 2007; Kangaroo Foundation, 2007), and in a recent state of the art report concerning NICU (Nyqvist et al., 2010). In the following text, we will use the term SSC to include all provision of this method to newborn infants, regardless of duration and hospital setting.

SSC is shown to have positive physiological, psychosocial, and developmental effects when provided for shorter or longer periods in the kangaroo position. Compared to traditional neonatal care, SSC/KMC has the same or a better outcome in heart and breathing rates, variations, and patterns; cerebral and body oxygenation; metabolism; temperature control; and growth and weight gain (Charpak et al., 2005). Meta-analyses have revealed improvement in mortality risk, incidence of nosocomial and severe infection/sepsis, duration of hospital stay, and hypothermia-risk (Conde-Agudelo et al., 2011). SSC has also been reported to reduce signs of pain during painful procedures (Pillai Riddell et al., 2011).

In infants with a low birth weight, meta-analyses have demonstrated improved rates of breastfeeding and exclusive breastfeeding, mother–infant attachment and interaction, parental and family satisfaction, and a better home environment (Conde-Agudelo et al., 2011); and in healthy newborns, breastfeeding frequency and duration were enhanced (Moore et al., 2007).

The growing implementation of SSC in neonatal care has brought a more active role for parents than previously possible in conventional care. This role involves new demands and responsibilities, but hopefully also benefits and positive experiences. As previously stated, three meta-analyses (Conde-Agudelo et al., 2011; Lawn, Mwansa-Kambafwile, Horta, Barros, & Cousens, 2010; McCall, Alderdice, Halliday, Jenkins, & Vohra, 2010) have been conducted on physiological outcome, but no previous qualitative meta-synthesis on parental experiences of SSC have been identified. Systematic reviews that generate theory by the interpretation of findings from primary studies are regarded as being vitally important for advancing nursing science. Furthermore, such reviews have a great potential to have a positive impact on policy and future practice (Noyes & Hayter, 2013; Paterson, Thorne, Canam, & Jillings, 2001). Within family-centered care, it has been argued that the focus should be both on the individual and the family system (Clarke, 1994), and thus it is important to understand the experiences of parents.

## The review

### Aim

The aim of the meta-synthesis was to synthesize and interpret findings from the body of research on parental experiences of SSC.

### Design

The present meta-synthesis was guided by the methodology described by Paterson et al. (2001). This approach includes analyzing the theories, methods, and findings in the studied qualitative literature; synthesizing the findings; and drawing new conclusions. Findings from each study are brought together to form a new perspective or theory. The methodology steps are as follows: 1) formulating a research question, 2) selecting and appraising primary research, 3) meta-data analysis, 4) meta-method, 5) meta-theory, 6) meta-synthesis, and 7) disseminating the findings (Paterson et al., 2001). Details of the three first steps are presented in part 1 (Anderzén-Carlsson et al., 2014).

### Search methods

The search includes published papers and doctoral dissertations up to November 2013 from the databases CINAHL, PubMed, SciElo, and LILACS. In addition, manual searches have been performed. The search methods, search outcomes, and quality appraisal have been described more in detail in part 1 (Anderzén-Carlsson et al., 2014).

### Search outcome

As presented in part 1 (Anderzén-Carlsson et al., 2014), the searches resulted in 320 papers, which were further appraised for eligibility and quality.

### Quality appraisal

In this step, the authors read and appraised the full text of the articles which were regarded as corresponding to the aim and inclusion criteria, and compared their decisions pertaining to inclusion or exclusion. Each paper was appraised by means of the Primary Research Appraisal Form presented by Paterson et al. (2001) by 2–3 researchers. The appraisal form is divided into sections describing the theoretical underpinnings, role, and credentials of researchers, as well as research design, method, and major findings.

The quality appraisal resulted in a final set of 29 papers forming the base for the present meta-synthesis.

### Data extraction

In the method described by Paterson, analyses of meta-data, meta-method, and meta-theory are performed. The meta-data analysis synthesizes data from the text of the included literature (Paterson et al., 2001). In the present study, data were retrieved from quotations and the researchers’ explicit findings. Qualitative content analysis (Graneheim & Lundman, 2004) was chosen as a strategy for the meta-data analysis. Although the meta-data analysis is one important foundation for the meta-synthesis, the details of the analysis and the detailed findings are presented in part 1 (Anderzén-Carlsson et al., 2014).

The meta-method phase consisted of analysis of how the original research methods and underlying assumptions influenced the results. Data for this analysis were extracted and presented in Table I, together with information about country, year, number of participants, and exposure to SSC. Where possible, we recorded demographic information about the parents and infants. Finally, the meta-theory phase aimed at exploring how the theoretical framework as well as political and sociocultural trends influenced the findings of the studies.

**Table I:** Studies included in the meta-synthesis.

Author (academic degree, profession), year of publication, country of study	Aim of the study	Theoretical orientation (T) Methodological orientation (M)	Population studied (P) Exposure to SSC (E)	Major findings
Affonso (RN, professor), Wahlberg (RN, professor), Persson (RN), 1989, Sweden	To identify and compare themes based on the reactions of two groups of mothers, using a cognitive adaptation framework.	T: Cognitive adaptation framework. M: Exploratory, descriptive design. Individual semi-structured interviews on the unit during and after the infant's care. Deductive content analysis using the attachment framework.	P: Sub-group of 33 healthy mothers, mean age 26.5 y (16–37 y). Total study: 66 mothers providing or not providing SSC. 33 infants, mean GA 31.1 w (26–28 w). E: When taken out of the incubator, the infants were healthy, stable and between 1 and 30 days.	According to the framework of the cognitive adaptation theory, the mothers searched for meaning and described a sense of mastery as well as self-enhancement.
Affonso (RN, professor), Bosque (RN, MS), Wahlberg (RN, professor), Brady (MD, neonatologist), 1993, USA	To explore the effects of KMC on mothers’ reactions	T: Cognitive adaptation framework. M: Individual semi- structured interviews. Deductive content analysis using the attachment framework.	P: 6 mothers (26–38 y). Infants GA 26–30 w, BW 765–1530 g in a NICU, Inclusion at 9–64 d. E: Minimum SSC 4 h/d, 6 d/w during 3 consecutive w.	SSC via the KMC method facilitates psychological healing and regaining the mothering role in an intensive care nursery.
Arrivabene (RN, MS), Tyrell (RN, professor), 2010, Brazil	To describe the mothers’ experiences of KMC, analyze them in the light of KMC principles, and discuss the mothers’ contributions based on the meanings of their experiences of KMC and thus implications for nursing practice.	M: Focus groups	P: 13 mothers (18–40 y) with low socioeconomic status in a NICU. No data on infants provided.	Increased bonding between mother and baby; reduction of the infant's separation from the family; increased competence and confidence on the part of the parents even before discharge; improved relationship between the mother and the rest of the family, within the family, and with the team taking care of the baby.
Blomqvist (RN), Hedberg Nyqvist (RN, assoc. professor), 2010, Sweden	To investigate mothers’ experiences of KMC.	M: Retrospective survey. Content analysis.	P: 10 mothers answered the open-ended questions. E: The total eligible group of 23 infants, GA 35.5 w, BW 2535 g, no data for the analyzed sub-group.	The mothers’ experiences were predominantly positive. Negative comments concerned lack of information. Some mothers perceived the care during the night as exhausting.
Blomqvist (RN), Rubertsson (midwife, assoc. professor), Kylbert (PhD, nutritionist), Jöreskog (PhD), Hedberg Nyqvist (RN, assoc. professor), 2012, Sweden	To describe fathers’ experiences of providing their preterm infants with KMC.	M: Semistructured interviews. Content analysis.	P: 7 first-time fathers, age 25–36 y. Preterm infants, GA 29–33 w, BV 1315–2500 g. E: KMC with father started day 1–5, between 211 and 478 min/d.	The fathers’ opportunity for being close to their infants facilitated attainment of their paternal role. They were active agents in their infant's care. The physical environment and conflicting staff statements influenced their experience.
Blomqvist (RN), Frölund (midwife), Rubertsson (midwife, assoc. professor), Hedberg Nyqvist (RN, assoc. professor), 2013, Sweden	To identify factors that parents of preterm infants perceived as supportive factors or barriers for their performance of KMC	M: Retrospective survey. Content analysis.	P: 76 mothers, 74 fathers Preterm infants GA 31.8 (28.4–33.9) w, BW 1781 (740–2920) g. E: The NICUs faciltated and encouraged KMC up to 24 h/d but no information on the included infants exporsure to KMC is provided.	Four categories were identified regarding support and barriers for parents’ performance of KMC: parent-related factors, time, infant-related factors, and the NICU and home environment. The hospital staff and environment were described by the parents as both supportive and barriers for their application of KMC.
Braga, Machado (RN, PhD), Bosi (Nutritionist, adj. professor), 2008, Brazil	To investigate perceptions and experiences of mothers of premature babies who breastfed exclusively from the 4th to the 6th month of life.	M: Individual open-ended interviews. Phenomenology.	P: 8 mothers aged 17–38 y. Infants GA<37 w, BW 1800–2500 g.	KMC is regarded as one of the factors that facilitate breastfeeding. One criterion is that the mother really wants to participate and has time, as the health care team must never impose the technique.
Byaruhanga (MD), Bergström (research assistant), Tibemanya (social scientist), Nakitto (midwife), Okong (MD, PhD), 2008, Uganda	To explore the perceptions of SSC among post-delivery mothers in order to identify factors that could influence the acceptability of this method.	M: Focus group discussions. Latent content analysis.	P: Sub-group of 30 mothers from another study, mean age 25 y, 18 multipara. Full-term infants. E: SSC after post-delivery bathing.	Acceptability of health practices influenced by knowledge and sensation. Pregnant women's choices dependent on social, cultural, and economic factors.
Caetano (RN, asst. professor), Scochi (RN, assoc. professor), Angelo (RN, professor), 2005, Brazil	To understand family dynamics and transformation as a result of KMC.	T: Symbolic interactionism. M: Grounded theory. Interviews with open questions.	P: 18 mothers in a KMC unit, mean age 27.2 y. Infants mean GA 29.2 w, mean BW 1195 g. E: 6–45 d in a KMC unit. with KMC all the time (no cots in unit).	The lived experience consists of one central category: Weighing the risks and benefits between staying with the child in the kangaroo method or with the family, involved three phenomena: 1) unexpected evolution and outcome in pregnancy, 2) coping with the prematurity of the child, 3) living with the decision and the experience together with the child.
Campos (RN, professor), Carvalho (RN), Rolim (RN, assoc. professor), Alencar (pediatrician, assist. professor), 2008, Brazil	To explore the mothers’ perceptions of KMC.	M: Descriptive study with qualitative approach. Semistructured interviews.	P: 13 mothers (19–39 y) in a KMC unit. No data on infants provided.	Strengthening of the bond between the mother and the newborn. Mothers recognize and appreciate the physical benefits for the infant and the opportunity to learn how to take care of a premature baby.
Dalbye (midwife), Calais (midwife), Berg (midwife, assoc. professor), 2011, Norway, Sweden	To explore experiences of SSC in healthy mothers of healthy, full-term infants in the first days after birth.	T: A lifeworld phenomenological approach. M: Interviewes. Phenomenology.	P: 3 primiparous and 7 multiparous women, age 24–37 y. Healthy full-term infants. E: First 2 h after delivery plus “as much as possible” in the first 24 h.	The SSC started a positive spiral. A mutual interaction developed which acted as a generator releasing energy to the mother. Happiness, peace, and satisfaction were expressed by the newborns.
Duarte (RN), de Sena (RN, assoc. professor), 2004, Brazil	To capture the mothers’ understanding of KMC and reveal the contradictions between the reality and their perceptions of the availability required to provide this care.	M: descriptive exploratory qualitative study, guided by the dialectical method.	P: 15 mothers in a KMC unit. No data on infants provided.	KMC is an opportunity to recover the disbanded unit, favoring transition from a pregnant woman to a mother. KMC is a form of process that involves women's bodies and emotions, strengthens their bond with the infant, and is perceived as rewarding.
Eleutério (RN), Rolim (RN, professor), Campos (RN, professor), Frota (RN, assoc. professor), Oliveira (RN), 2008, Brazil	To explore the perceptions of mothers who experienced KMC during hospitalization in the KMC infirmary.	M: Semi-structured interviews. Content analysis.	P: 9 mothers in a KMC unit. No data on infants provided.	Four themes: 1) knowledge, 2) care, 3) receptivity, 4) caress. The mothers considered the Kangaroo method an opportunity for learning how to care for their babies and that bonding is relevant and helps in the baby's recovery.
Finigan (MSc, midwife), Davies (MPhil, midwife), 2004, England	To explore women's lived experiences of SSC with their baby immediately after birth. To investigate the experiences from the women's own perspective and establish whether or not this is a mother-friendly approach.	M: Audio diaries from birth up to 28th day postpartum. In-depth interviews. Thematic analysis.	P: 6 mothers (21–36 y), 5 multigravidas. E: SSC within 30 m of the birth and maintained for at least 1 h.	Five themes: 1) immediate feelings of bonding, 2) touch and stroking, 3) the gaze and getting to know the baby, 4) natural, instinctive behavior, 5) not wanting to let go of the baby.
Furlan (RN, MSc), Scochi (assoc. professor), Furtado (MSc), 2003, Brazil	To analyze preterm babies’ parents’ perceptions of KMC, in order to introduce subsidies for the promotion of humanized assistance to support NICU clients.	M: Qualitative descriptive. Semi-structured interviews 60 days after discharge from the KMC-unit. Thematic analysis.	P: 5 couples (5 mothers, 5 fathers) (18–33 y). Preterm infants, about 35 w and 1100–1500 g at start of KMC. E: KMC 8–12 h/d for 12–30 d.	Four thematic nuclei: 1) The flexibility of the maternal stay in the KMC ward, 2) giving support to mother-child and family relationships, 3) completing the growth and development of the premature infant, and 4) developing skills to take care of the premature baby.
Heinemann (RN), Hellström-Westas (neonatologist, professor), Hedberg Nyqvist (RN, assoc. professor), 2013, Sweden	To describe parents’ experiences of factors that influenced their stay with their extremely preterm infants in an NICU.	M: Qualitative descriptive. Semi-structured interviews at least 1 w after transfer to home or step-down unit. Content analysis.	P: 7 couples (7 mothers, 6 fathers). Preterm infants GA 23 w+5 d to 27 w+6 d, BW 492–1044 g. E: not specified but SSC for all infants was facilitated and encouraged at the NICU.	Two themes: 1) coping with a new and unexpected situation, and 2) ecoming a parent.
Helth (RN), Jarden (RN, PhD), 2013, Denmark	To explore how fathers of premature infants experience and potentially benefit from using skin-to-skin method during the NICU stay.	M: Hermeneutic phenomenological. In-depth, semi-structured interviews.	P: 5 first-time fathers (28–37 y). Preterm infants GA<35 w. E: not specified but all fathers had SSC experience at the NICU.	Three themes: 1) the competent parenthood, 2) the paternal role and the division between the parents, 3) balance between working life and time spent with the infant.
Johnson (RN, assoc. professor), 2007, USA	To describe mothers’ experience of kangaroo holding of premature infants in the neonatal intensive unit as a means of gaining insight into specific maternal benefits of this intervention.	T: No explicit theoretical orientation. In the Discussion section, maternal role, development, and attachment are elaborated on. M: Qualitative naturalistic inquiry. Open-ended interviews. Content analysis. Observations were also carried out, the results of which were combined with the interview data.	P: 18 primiparous mothers, mean age 26.3 y. Infants mean GA 28.8 w, mean BW 1410 g. E: Held infant for 60 min in the NICU on three occasions during the first 2 weeks of her/his life.	Three themes: 1) maternal–infant benefits of kangaroo holding, 2) need of support for holding, and 3) satisfaction with interaction.
Lamy (neonatologist, professor), Morsch (psychologist), Deslandes (sociologist, assoc. professor), Fernandes (neonatologist), Moreira (neonatologist), Gomes (MD, assoc. professor), 2011, Brazil	To reveal how women construct their maternal role when they have had a preterm and/or low birth weight infant in an NICU.	M: Semi-structured interviews.	P: 20 mothers from 4 hospitals, having their infant for 1–3 m in the NICU. No data on infants provided.	KMC helped the women to feel like and consider themselves mothers. They also expressed confidence in the baby and felt more certain in their maternal role.
Leonard (RN, MScN), Mayers (MScMed), 2008, South Africa	To explore the lived experiences of parents who provided their preterm infant with KMC.	M: A qualitative, explorative and contextual study in the phenomenological tradition. In-depth interviews.	P: 4 mothers, 2 fathers. Premature infants BW>1000 g,>1 w. E: Active provision of KMC in the neonatal nursery and KMC ward of a tertiary hospital.	Six themes: 1) unforeseen, unprepared and uncertain—the experience of birth, 2) anxiety and barriers, 3) an intimate connection, 4) adjustments, roles and responsibilities, 5) measuring success, and 6) a network of encouragement and support.
Martins (RN), Santos (assoc. professor), 2006, Brazil	To identify the mothers’ difficulties participating in KMC and observe the strategies they used to overcome the difficulties.	M: Qualitative descriptive design. A structured questionnaire with five open questions. Interviews. Thematic analysis.	P: 5 mothers (17–34 y) participating in KMC in an Intermediate Care Unit. Infants GA <37 w, BW <1250 g. E: KMC as soon as the infant was medically stable, continuously if possible.	The thematic analysis resulted in two categories: 1) learning how to be a kangaroo mother, 2) living as a kangaroo mother.
Moura (professor of psychology), Araújo (PhD, psychologist), 2005, Brazil	To understand KMC users’ perceptions of the concept of motherhood and their motherhood experience.	M: semi-structured interviews and observations. French Discourse Analysis based on Foucault and Guattari's notion of subjectivity.	P: 8 low-income mothers in hospital KMC unit. No data on infants provided. P: 8 low-income mothers in hospital KMC unit. No data on infants provided. E: Holding baby in kangaroo position.	1) family and religion were characterized as central elements in attribution of meaning to motherhood, 2) the impact of premature birth, leading to disruption in the construction of the maternal role, 3) relationships with institutions and health professionals characterized by distrust and resistance, and 4) experience of KMC, which provided an opportunity to establish contact with the child and gain confidence in one's own mothering role.
Nakajima (RN, PhD), 2002, Japan	To study the effect of kangaroo care on maternal attachment and healing.	M: Comparison analysis. Triangulation approach was used to compare the similarities and differences of the qualitative and quantitative results. Interviews analyzed by means of comparison analysis.	P: 20 mothers who experienced KMC on more than three occasions. Premature infants BW<2500 g. E: KMC after an infant reached GA 32 w, up to 2 h/d.	Three themes: 1) feelings of guilt and uncertainly were alleviated, 2) mothers felt released from the constant feeling of hurt or pain, and 3) obtained a greater sense of “this is my child.”
Neu (RN, PhD), 1999	To explore parents’ perceptions of providing SSC to their preterm infant who was receiving assisted ventilation and elucidate factors that influenced the decision to continue or discontinue SSC.	M: Naturalistic inquiry. Interviews. Content analysis.	P: Sub-group of 8 mothers and 1 father from another project, mean age 25.9 y (21–37). 4 primiparous. Premature infants, mean BW 1064 g (SD 423), mean GA 27.2 (2.0) w. E: Two 60 m SSC sessions on consecutive days.	Three themes: 1) ambivalence of parents toward SSC, 2) need of a supportive environment, and 3) special quality of the parent–infant interaction.
Neu (RN, asst. professor), 2004, USA	To describe factors that influenced mothers of healthy preterm infants to choose kangaroo holding rather than the standard blanket holding method.	M: Naturalistic inquiry. Interviews. Content analysis.	P: 24 primiparous mothers, median age 30 y (18–41). Healthy infants, median GA 32.5 w (31–34). E: The KMC regimen was standard in the NICU and also provided after discharge.	Three themes: 1) expression of emotional distress, 2) perception of a facilitative environment for holding, 3) perceived benefits of close contact with the infant.
Neves (RN), Ravelli (MSN), Lemos (master degree), 2010, Brazil	To identify mothers’ perceptions of KMC.	M: Semi-structured interviews.	P: 6 mothers in a KMC unit. Preterm, stable infants.	KMC made the mothers more familiar with their infant.
Roller (midwife, asst. professor), 2005, USA	To gain an understanding of mothers’ experiences of providing KMC for their preterm newborns.	T: Maternal–infant attachment theory. M: Transcendental phenomenology. Semi-structured interviews, observations.	P: 10 mothers. Preterm infants, GA 32–26 w, BW 1500–3000 g. E: KMC within the first 24 h after birth at the neonatal unit.	Four main themes, which were reduced to one essential structure of knowing: mothers were prevented from knowing or getting to know their preterm newborn. Only one theme concerned the aim of our study; Kangaroo Care and also some parts of the theme Reassurance.
Toma (pediatrician), 2003, Brazil	To increase understanding of the influence of hospital conditions and family organization on KMC practice.	M: Qualitative descriptive design. Interviews based on a guide.	P: 14 young mothers (10 first time) and 7 fathers. No data on infants provided.	The opportunity for effective parent participation from the beginning of life supports the creation and strengthening of the relationship and makes taking care of the child easier. However, the success of KMC does not only depend on the mothers’ will but also on the support of family networks and of a comprehensive health care staff.
Toma (pediatrician), Venancio (MD, PhD), Andretto (psychologist), 2007, Brazil	To improve KMC by contributing to the knowledge of the different ways in which low-income families deal with a preterm baby.	M: Grounded theory. 2 interviews, on day of discharge and at home after 15–30 d. 3 open questions about pregnancy, childbirth, as well as hospital and home postpartum periods.	P: 22 mothers, mean 26 y. (part of a larger sample containing an additional 19 mothers pre-intervention). Infants BW<2000 g who remained>1 w at the NICU.	The need to care for their other children appeared to be one of the main KMC constraints. The trend toward nuclear families hindered women's participation in the program. Awareness of the limitations and possibilities of each family may contribute to improved implementation.

KMC=kangaroo mother care; SSC=skin-to-skin care; BW=birth weight; GA=gestational age; y=year; w=week; m=month; d=day; h=hour; min=minute; g=gram; RN=registered nurse; MS=master of science; MD=doctor of medicine; NICU=Neonatal Intensive Care Unit

### Synthesis

The results of the three analysis phases are presented in the Results section. In the final step, the meta-synthesis, insights from each of the three analyses were brought together in a theoretical model. Because of the amount of details in the findings based on the meta-data analysis, these findings are only presented in a summarized way, and are presented in more detail in part 1 (Anderzén-Carlsson et al., 2014).

## Results

**Figure 1:**
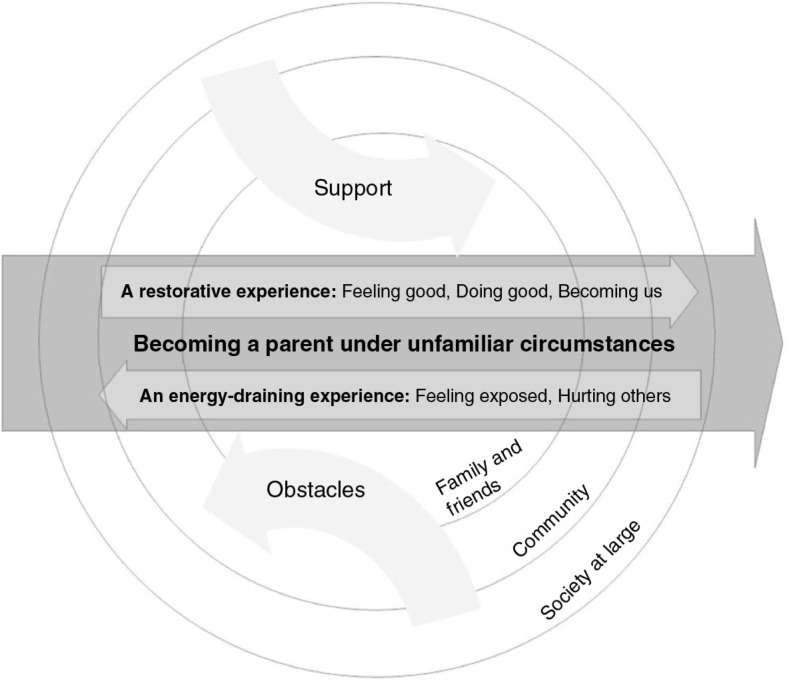
A model of “Becoming a parent under unfamiliar circumstances.”

*Becoming a parent under unfamiliar circumstances* is a theoretical model based on the meta-synthesis of the results from the 29 papers on parents’ experiences of providing SSC to their newborn infant. Providing SSC was seen as both a restorative and an energy-draining experience. The premature birth with subsequent NICU care, or a post-delivery phase that differed from a previous experience, formed the base for the interpretation of the circumstances being unfamiliar for the parents. The process of becoming a parent through the SSC was influenced by external factors from three different societal levels: family and friends, community, and society at large. A supportive environment was described as facilitating the restorative experience, whereas obstacles in this environment seemed to make the provision of SSC energy-draining for parents. When the process was experienced as positive, it facilitated the growth of parental self-esteem and made the parents ready to assume full responsibility for their child. These patterns lead to our theoretical model, as illustrated in Fig. 1. The findings showed a similar pattern to what previous researchers have described as central features in their theories of becoming a mother or father. These underpinnings for the model will be further highlighted in the Discussion section.

### Results from the meta-data analysis

The detailed results of the meta-data analysis are presented in part 1 (Anderzén-Carlsson et al., 2014). Thus, only a short summary is provided here, for the reader to follow the reasoning behind the interpretation of the findings resulting in the theoretical model *Becoming a parent under unfamiliar circumstances*. An overview of the themes, sub-themes, and categories and their relation to the included articles is provided in Table II.

**Table II:** Overview of the identified themes, sub-themes, and categories.

	A restorative experience													An energy-draining experience					
	
	Feeling good									Doing good		Becom-ing us		Feeling exposed				Hurting others	
	
	A heart-warming experience	Relieving emotional suffering	A rewarding experience	A natural instinct	A learning experience	Finding a role	Improved self-esteem	Feeling of control	A supportive environment	A way of knowing and understanding	Important for the infant	A bonding experience	Intimate togetherness	Environment as an obstacle	The physical and emotional burden	Incongruence between wishes and demands	Uncertainty about the purpose of and own skill in providing SSC	Fear of hurting	Feeling insufficient toward the family
Affonso et al. 1989	X	X		X	X	X	X	X	X	X		X					X		
Affonso et al. 1993	X				X	X	X	X		X	X	X	X		X	X		X	
Arivabene & Tyrell 2010									X		X	X	X			X			X
Blomqvist & Nyqvist 2010							X		X					X	X	X			X
Blomqvist et al. 2012						X			X	X	X			X	X				X
Blomqvist et al. 2013									X	X	X				X	X		X	X
Braga et al. 2008		X			X						X	X		X	X			X	
Byaruhanga et al. 2008	X			X				X			X	X		X		X		X	
Caetano et al. 2005		X		X					X		X		X			X			X
Campos et al. 2008	X	X	X		X	X	X	X	X		X	X							X
Dalbye et al. 2011	X		X								X	X	X	X				X	X
Duarte & de Sena 2004			X					X			X	X			X				X
Eleutério et al. 2008					X	X	X			X	X	X	X	X			X	X	X
Finigan & Davies 2004	X		X	X		X	X				X	X	X			X			
Furlan et al. 2003	X	X			X	X	X	X	X	X	X	X	X	X	X	X			X
Heinemann et al. 2013	X					X			X				X	X		X			
Helth & Jarden 2013	X				X	X	X	X			X	X		X		X		X	
Johnson 2007	X					X	X			X	X	X	X			X	X	X	X
Lamy et al. 2011	X				X	X	X		X	X	X	X			X				X
Leonard & Mayers 2008	X				X	X	X		X	X	X	X	X	X	X	X	X	X	X
Martins & dos Santos 2006					X		X		X		X				X	X	X	X	X
Moura & Araújo 2005	X	X	X			X	X	X			X	X		X	X	X	X		
Nakajima 2002		X					X			X	X	X	X		X			X	
Neu 1999				X		X	X		X		X	X		X	X	X	X	X	
Neu 2004	X								X	X	X	X	X	X	X	X		X	
Neves et al. 2010	X							X	X	X	X	X		X	X			X	
Roller 2005	X					X	X	X			X	X		X	X				
Toma 2003					X	X		X	X	X	X	X	X	X	X	X	X	X	X
Toma et al. 2007					X			X	X		X	X		X	X	X		X	X

### A restorative experience

#### Feeling good

Feeling good is a restorative experience that refers to the parents’ experience of well-being associated with their role as a mother or father or as an individual. It includes an immediate and intimate sense that SSC is natural and rewarding for parents. The parents described fascination over the infant's competence and development. Furthermore, it is a learning experience connected with finding a role and enhanced self-esteem, preparing the mothers to assume full responsibility after discharge from the hospital. SSC also increases parents’ well-being by reducing emotional pain and strengthening their hope for the infant's recovery. A supportive environment is described as important for encouraging parents to provide SSC. Support can be from professionals, family members, or other parents in the same situation, but the physical environment or religious faith can also facilitate SSC.

#### Doing good

Doing good is also a restorative experience in which parents described SSC as primarily beneficial to someone other than themselves and yet of personal value to them. It is a way of getting to know the newborn infant, providing protection, participating in her or his recovery, and offering themselves to the infant, thereby being of importance for her or him.

#### Becoming us

Parents described SSC as having a relational component. By a reciprocal process, the parents and infant came to know each other, bonded, and became attached. The parents also experienced a strong intimate togetherness not only with the infant, but also as a couple and family.

### An energy-draining experience

#### Feeling exposed

From the analysis, it appeared that parents experienced themselves as being in an exposed situation when providing SSC. These feelings were based on external factors, such as an environment that was as an obstacle. Environment included both the staff and the physical milieu, for example, lack of privacy. Internal factors that contributed to feeling exposed were uncertainty about their own SSC skill and the sense of hurting other family members by providing SSC. Another reason for feeling exposed was the tension between the wishes and demands on the parents, who experienced both a physical and an emotional burden when providing SSC. At times, mothers described that they felt restricted by the SSC. On the contrary, fathers who were prevented from providing SSC for organizational reasons were frustrated and helpless, as they could not interact with their infant. In two of the Latin American studies, parents highlighted that travel associated with SSC was a financial burden. Although feeling exposed by the SSC, it was regarded as important.

#### Hurting others

Hurting others is an energy-draining experience of providing SSC that was part of the process of becoming a parent and comprises two perspectives. First, being afraid of hurting the infant, irrespective of whether she or he was premature or full term and, second, SSC creates feelings of insufficiency toward other family members, especially older siblings.

### Results from the analyses of meta-theory and meta-method

The included individual studies revealed parents’ experiences of providing SSC in the light of theory or for a specific objective as presented in Table I. Because of the different aims, it is understandable that the scopes of the findings vary, from a specific to a more general description. The previous results (major findings, themes, etc.) are all in line with the findings of the present study, although in the original publications they often just illustrate one single piece of a large jigsaw puzzle; one aspect of a more comprehensive picture.

Most of the studies were performed at a time when there was a positive interest worldwide in implementing SSC, which may have had an impact on the findings, as almost all of the individual studies highlight the benefits of SSC. However, the meta-data analysis made the drawbacks from a parental perspective more visible and distinct. In the early SSC-research, findings about parental needs of support were more seldom explicitly expressed, and only in a recent article are parental concerns highlighted as an important issue (Thernström Blomqvist & Nyqvist, 2010).

The findings of this meta-synthesis have a universal pattern, irrespective of whether they are based on studies of normal deliveries or experiences of providing SSC to a premature infant (Table I). Most of the participants in the studies were mothers (*n=*401) and there were 94 fathers, mainly in the most recent papers. In view of this fact, the outcome of the meta-synthesis is strongly related to mothering and motherhood, but as fathers were included, we have agreed to use the term parent instead of mother in our theoretical model.

An analysis of the data collection methods (Table I) in relation to the identified sub-themes did not reveal any obvious pattern. Most studies were represented in all sub-themes, although three of the studies using semi-structured interviews did not present findings related to hurting others by providing SSC (Affonso, Wahlberg, & Persson, 1989; Moura & Araújo, 2005; Roller, 2005). This may be because of the predefined topics in these interviews.

The professional categories of the researchers are presented in Table I. Three of the midwife-driven studies focused on the SSC experience in relation to delivery (Byaruhanga, Bergstrom, Tibemanya, Nakitto, & Okong, 2008; Dalbye, Calais, & Berg, 2011; Finigan & Davies, 2004), but their findings are in line with the other studies. Apart from the three studies performed in a delivery ward setting, the majority were conducted in a NICU context (*n=*15), a KMC-unit (*n=*8), or not specified (*n=*3) (Table I). All studies conducted in KMC units originated from Latin America. The setting does not seem to have had a major impact on the results.

## Discussion

Previous research has revealed various, mainly positive, experiences of providing SSC. This meta-synthesis of 29 original papers led to a new and more complex, yet comprehensive, picture; the experience of becoming a parent under unfamiliar circumstances. The biological parents seem to enter into parenthood by a process awakened by the skin-to-skin interaction with the infant. In the following text, our findings will be discussed in relation to the literature on becoming or being a mother or father. Most of the literature identified seems to focus on becoming a mother (Holm, 1993; Meighan, 2006; Mercer, 1995, 2004; Rubin, 1984), but some studies took the perspective of becoming a father (Andersen, 2003; Fagerskiold, 2008; Premberg, Hellstrom, & Berg, 2008). It is often emphasized that becoming a mother or father is something created in relation to someone else, not preprogramed behavior (Andersen, 2003; Mercer, 1995, 2004; Rubin, 1984), which corresponds with the interpretation of the findings in this meta-synthesis.

Rubin (1984), pioneer of maternal identity and experience research stated “The act of giving birth is not completed for a woman until she can hold her baby” (p. 108). This statement implies that holding the baby is of great importance for the woman who has given birth. In the findings of this meta-synthesis, it also appears that SSC was important for the mothers and made them feel good. They described it as rewarding and natural. Their fascination about the child and her or his ability connected to the experience of SSC is similar to what Rubin (1984) described as sensing the miracle of life, its wonder and beauty forms a matrix of bonding that is unique to human females and related to giving birth. Rubin's studies of women becoming mothers demonstrate that giving birth to a baby is connected to spiritual, religious, and philosophical experiences. In our results, such perspectives were labeled a heart-warming experience and were, to some extent, linked to SSC being a reward to the mother. Mercer, one of Rubin's fellow scholars, described the latter as gratification, which in Mercer's model is part of the process of becoming a mother (Mercer, 1995).

Mercer (2004) identified four, somewhat overlapping, stages in the process of becoming a mother, from pregnancy to approximately 4 months postpartum. She defined the first stage, *Commitment, attachment, and preparation*, as occurring during pregnancy and states that the woman's active involvement in this phase is linked to a positive adaptation to motherhood. However, according to the present findings, this stage seems to happen during the premature infant's hospital care period. Our findings provide evidence of SSC increasing the parents’ commitment to the infant. They described SSC as a heart-warming experience and were fascinated by the infant's competence and also at times by the fact that they were able to see her or his development. Similarly, research on becoming a father has revealed that fathers were overwhelmed and that a newborn infant brings warmth and happiness into the family (Fagerskiold, 2008; Premberg et al., 2008). Likewise, it has been demonstrated that fathers find it exciting to follow their newborn infant's development from the first moment (Premberg et al., 2008). In terms of *preparation*, it was also apparent in the findings that SSC helps parents to prepare themselves to assume full responsibility for the infant and to leave the hospital.

The second stage, *Acquaintance, learning, and physical restoration*, generally occurs during the first 2–6 weeks after delivery (Mercer, 2004). Based on the findings, it is possible to conclude that, in terms of SSC, this stage is described as a way for the parents to get to know and understand the infant and be of importance for her or him. It is also described as a learning experience, a feeling of control, and finding a role, which, from the parents’ perspective, leads to improved self-esteem. Similar outcomes have been revealed during the initial year as a first-time father (Premberg et al., 2008). Finally, this phase is represented by statements regarding the bonding experience, where the mothers and fathers bond with the infant and vice versa. Unlike in Mercer's theory (1995, 2004), the physical restoration of the mother is not highlighted in this meta-synthesis, which could be because of the difference in focus. However, in the results, a psychological restoration is described in terms of release from hurt and pain.

The third and fourth stages, *Moving toward a new normal (2 weeks to 4 months)* and *Achievement of the maternal identity (around 4 months)* (Mercer, 2004), can be considered related to the results describing that, because of SSC, the mothers and fathers experienced themselves as parents, felt more secure about their SSC skills, and reported increased self-esteem, thereby making them ready to assume full responsibility for the infant at discharge (which can take some months, depending of degree of prematurity and illness). In the first of these phases, Mercer (2004) identified that the mother adjusts to the changes in relationship with her partner, which is similar to the findings on “becoming us.” In the results, it is not always possible to specifically trace the process of becoming a parent, as the data in the various studies have been collected at different points. Mercer, however, claimed that there could be an overlap between the stages (Mercer, 2004); thus, we believe it is possible to apply the various findings in the process that she identified.

In her theoretical work on *becoming a mother*, Mercer described the process as influenced by three levels: *family and friends, community*, and *society at large* (Meighan, 2006). In the present findings, it is possible to trace the influence of all three, but especially the first two, which is partly in line with Mercer's theory, where she states that the level of family and friends is the most influential in becoming a mother (Meighan, 2006; Mercer, 1995). The impact of the nuclear as well as the extended family and friends (both experiences of support and feelings of abandoning/hurting the family) identified in the findings can be regarded as the *family and friends level*, whereas experiences related to the hospital environment and staff can be seen as equal to the *community level*. Findings that can be interpreted as related to the impact from the *Society at large* level were the financial consequences of having to travel back and forth to the hospital in order to provide SSC (Brazilian context) and the benefit from the government providing social benefits that allowed parents to take a leave from work to be with their infant in the NICU and provide SSC (Swedish context).

As previously stated, it was revealed by the meta-synthesis that parents experienced that they got to know the infant by providing SSC, for example, by learning to recognize her or his signals. Andersen (2003) holds that being a father is more of a social construction than purely biological, which relates to the interpretation that getting to know the infant is linked to becoming a parent. He stated that fathers need to become aware of the child's needs and signals to experience the appeal of becoming a father and pointed out that contact with the child is one important factor for entering into fatherhood. The philosophical explanation is that as the child learns to know or recognize the father, the child becomes a subject in the father's eyes. The subject talks to or communicates with the father in a unique way that appeals to the father (Andersen, 2003). Likewise, the mother needs interaction with the infant as, according to Rubin (1984), maternal behaviors are uncertain without feedback from the child. Based on this reasoning, it can be assumed that the interaction experienced by the parents when they learn parenting skills and behavior by means of SSC are aspects of becoming a parent, which enhances their self-esteem.

As the parents and infant begin to know each other, they bond and attach. Holm, a Swedish philosopher, coined the concept “mothering” [“modrande”] (Holm, 1993). She stated that mothering is an interactive, ongoing, and changeable relationship that is asymmetric, yet reciprocal. This relationship is often biological, but does not have to be so. Instead the relation is based on an I-and-you relation between a helpless child and a person that enters into a long-lasting caring relation with that child. The mother does not know the child at birth, but is eager to get to know her or him. Mothering is related to “doing” things, which, based on the findings of the meta-synthesis, could be exemplified by providing SSC as such, or the secondary “effects” that occur as a consequence of SSC, such as monitoring or caring for the infant. Holm emphasized that fathering can be regarded as similar to mothering, but did not elaborate on it in her work, although she questioned the assumption of the symbiosis between a child and *one* adult. Premberg et al. (2008) found that spending time alone with their offspring enhanced the contact between first-time fathers and their infants, and over time the fathers learned to recognize their infant's signals, similar to the findings from the meta-synthesis.

The experience of becoming us includes not only the parent–child relationship but also an intimate togetherness between the parents. This has similarities with previous findings on experiences of the first year of fatherhood. Being part of a family has been described as entering into a new and deeper wholeness in life (Premberg et al., 2008), where the relationship with the mother is strengthened (Fagerskiold, 2008; Premberg et al., 2008). In the theoretical literature, it has been stated that formation of a maternal identity and the performance of maternal tasks are influenced by the husband–wife relationship and that there is an association between the strength and function of a mother's intra-family bonds and the quality and strength of her bond with the child (Rubin, 1984). In the studies that focused on family functioning when providing SSC, it was found that the fathers “helped” with household tasks or “helped” the mother so that she could provide SSC. This can be understood as a cultural aspect of fathering where, according to Holm (1993), one responsibility is to enable conditions in which the mother can provide mothering. It can also be related to support, which according to Mercer (1995) is one factor that has an impact on becoming a mother. Studies focusing on becoming a father have found that first-time fathers viewed themselves as supporting the mother (Fagerskiold, 2008; Premberg et al., 2008). In line with the reasoning of the correlation between the strength and function of a mother's intra-family bonds and the quality and power of her bond with her child (Rubin, 1984), it is reasonable to believe that the husband's or partner's support in the SSC-situation can facilitate better maternal bonding, whereas lack of support might consequently lead to more fragile bonding.

The findings about being important for the infant and especially protecting the infant correspond with what Holm (1993) described as the first and most obvious demand in mothering. These results also appear to be related to the findings of being afraid of hurting the infant. Holm stated that the mothering person is aware of the unpredictable consequences of mothering, as well as the fragility of life. According to Holm, another demand inherent in mothering is to promote the child's development, which is similar to the findings about being important for the infant's recovery.

In addition to the restorative experience, this meta-synthesis found SSC to be energy-draining. A sense of hurting others or being afraid of so doing when providing SSC constituted such an experience. This finding was multidimensional; on the one hand, the parents were afraid of hurting the infant, which can be related to anxiety, a factor that Mercer (Meighan, 2006) identified as part of becoming a mother. On the other hand, a sense of imbalance in terms of loyalty was experienced, especially to the infant's older siblings, something identified by Mercer (1995) as well. Rubin (1984) more specifically identified a sense of guilt in relation to older children experienced by mothers of a newborn and stated that they must make a great effort in order to maintain and promote the well-established bonds with the older children.

Although feeling tied to the ward and the infant when providing SSC, parents nevertheless chose to provide it, as they realized that it was good for the infant. In the literature on becoming a father, it has been acknowledged that fatherhood can be associated with a wish for independence (Andersen, 2003; Premberg et al., 2008). Premberg et al. (2008) therefore stressed that first-time fathers should have their own leisure activities to promote their well-being and adaptation to their new situation. Rubin (1984) stated that the sacrifice related to becoming a mother is already substantial during pregnancy. According to Holm (1993), mothering is often connected to strong and conflicting emotions, similar to the pattern found in the meta-synthesis.

Furthermore, in the findings parents were found to feel insecure when caring for the infant. In Andersen's (2003) studies of fatherhood, uncertainty about one's own capacity to assume the responsibility of being a father was identified. In Andersen's (2003) work, this feeling was described in relation to the child's mother, but in our meta-synthesis the parents did not express much uncertainty in relation to the other parent, but rather to the professionals taking care of their infant. They may have regarded the professionals as the primary caregiver, which supports the idea that a feeling of insecurity toward the primary caregiver could be a pattern of entering into parenthood.

The energy-draining elements of parenthood, that is, fear of hurting others and the feeling of being exposed identified in the meta-synthesis, are not especially highlighted in Mercer's (1995, 2004) theoretical work. It is likely that these feelings are stronger in the vulnerable group of parents of premature or sick children and therefore more clearly expressed in such a context (Obeidat, Bond, & Callister, 2009). However, Mercer recognized that stressors can influence the process of becoming a mother, in the same way as support, cultural guidelines, and family values or functioning (Mercer, 1995). In this meta-synthesis, such factors were described as having an impact on the parents’ feeling of being exposed but also as being beneficial. The positive experiences of a supportive environment, as well as the negative ones, when the environment constitutes an obstacle, can therefore, according to Mercer's model, be related to the community environment (Meighan, 2006). In their work on becoming a first-time father, Premberg et al. (2008) reported that fathers sometimes felt ignored when participating in childbirth education programs, as the mother and her needs were in focus. Similar feelings were found in the meta-synthesis, where fathers wanted to provide SSC for their infant at the delivery ward or expressed a wish to practice more SSC than they were allowed at the hospital. To conclude, previous research and theoretical work on becoming a parent support the theoretical model identified by the meta-synthesis.

### Limitations and strengths

The aim with conducting a meta-synthesis is not to question the results in the included studies, nor to merely aggregate previous findings. Instead, it is to generate a more complete understanding of the phenomena under study. The method used has an interpretive constructive approach (Paterson et al., 2001). The authors have, during the entire research process, had the ambition to be truthful to the included material, and interpretations at all levels have continuously been discussed among the authors.

In the first database search, a large body of studies published in languages other than English were identified. This led us to expand our search to two Latin American databases and to include findings from papers written in Japanese, English, and Portuguese in the analysis. As we set no time limit, this study comprises all published research about parental experiences of SSC until November 2013, as far as we are aware of. The choice to include studies from the delivery care department, where mothers provided SSC to healthy newborns, can be regarded as a limitation with regards to generalizability of the findings. However, these results were regarded as being in line with the findings from the other included studies and can thereby be seen as confirming the identified model. Furthermore, it is possible to question the decision to include both mothers’ and fathers’ experiences in the same meta-synthesis, because the majority of the original data are based on mothers’ experiences. In the discussion section, we have tried to justify this decision by comparing the findings from the meta-synthesis with literature on becoming a mother, as well as a father.

Not all studies presented the necessary data for the meta-method part of the analysis. When possible, we contacted the authors and obtained additional information. Two or three researchers participated in all steps (selection, appraisal, data extraction, and analysis) of this study. The use of nVivo facilitated the analysis phase by providing a good overview and supporting the formulation of categories and sub-themes.

By including original articles covering large geographical and cultural areas, as well as most published research until 2013, it is reasonable to believe that the results can be generalized to a broad parental population providing SSC to their newborn infant in hospital setting.

## Conclusion

The knowledge that parental provision of SSC has implications for the family beyond the actual SSC-situation is not new. However, the fact that such provision can be seen as becoming a parent under unfamiliar circumstances has not previously been described. From a family-centered perspective, it seems important to take this fact into consideration, as some of the situations described could threaten the future of the infant and family. When providing family-centered care, health care professionals should focus simultaneously on the individual and the family system (Clarke, 1994). It seems important to take the results of this meta-synthesis into consideration when caring for families who practice SSC, as the parents report both restorative and energy-draining experiences. When providing professional care, it is easy to focus on known benefits for the infants and parents, but we emphasize that the whole picture must be taken into account. The parents, mainly mothers, experienced that SSC made them feel good, that it was a beneficial thing to do for their baby, and helped them to bond with their infant. Although they experienced a supportive environment that facilitated SSC, they at times felt exposed and described the environment as an obstacle. It seems to be of importance for professionals to be aware of these contradictory aspects of the environment and become aware of each individual parent's experience. Mercer (1995) emphasized that the kind of care a new mother receives during the first year following birth can have long-term effects on her and her offspring. She argued for nurses being responsible for families’ and children's health, including assessment of the family situation and environment and setting goals with them. One such short-term goal can be to maintain contact with older children, as the strain of dividing oneself between the infant and older children has been described as burdensome. Furthermore, the nurse should assist the family by teaching, supporting, and providing care that the client is unable to do herself. Health care providers can offer emotional, informational, physical, and appraisal support (Meighan, 2006), all of which have been found to be important for parents providing SSC. Furthermore, when planning new neonatal units, it seems important to allow for the provision of SSC by both parents, for example, by furnishing in a way that facilitates privacy and physical comfort. Professionals should also be aware of how they can facilitate SSC for parents, for example, by making it easier for them to eat or take a break from the responsibility of providing SSC without feeling guilty.

In future studies it would be interesting to specifically investigate fathers’ experiences of providing SSC, as well as investigating SSC from a family-centered perspective as opposed to only focusing on one actor at a time. Yet another field for future studies would be to test the relevance and applicability of the identified theoretical model in clinical practice and future research.
